# Adoption and Use of Telemedicine and Digital Health Services Among Older Adults in Light of the COVID-19 Pandemic: Repeated Cross-Sectional Analysis

**DOI:** 10.2196/52317

**Published:** 2024-04-24

**Authors:** Motti Haimi, Ruslan Sergienko

**Affiliations:** 1 Rappaport Faculty of Medicine Technion-Israel Institute of Technology Haifa Israel; 2 Health Administration Department The Max Stern Yezreel Valley College Emek Yezreel Israel; 3 Clalit Research Institute Tel Aviv Israel; 4 Department of Health Policy and Management Faculty of Health Sciences Ben-Gurion University of the Negev Beer Sheva Israel

**Keywords:** telemedicine, digital health, older adults, COVID-19, use, digital divide, usability, pandemic, telehealth, Israel, working-hours telehealth visits, after-hours consultation, teleconsultation, eHealth, mobile health, mHealth, wearables, mobile phone

## Abstract

**Background:**

As the population ages and the prevalence of long-term diseases rises, the use of telecare is becoming increasingly frequent to aid older people.

**Objective:**

This study aims to explore the use and adoption of 3 types of telehealth services among the older population in Israel before, during, and after the COVID-19 pandemic.

**Methods:**

We explored the use characteristics of older adults (aged ≥65 years) belonging to Clalit Health Services in several aspects in the use of 3 types of telehealth services: the use of digital services for administrative tasks; the use of synchronous working-hours telehealth visits with the patient’s personal physician during clinic business hours; and the use of after-hours consultations during evenings, nights, and weekends when the clinics are closed. The data were collected and analyzed throughout 3 distinct periods in Israel: before the COVID-19 pandemic, during the onset of the COVID-19 pandemic, and following the COVID-19 peak.

**Results:**

Data of 618,850 patients who met the inclusion criteria were extracted. Telehealth services used for administrative purposes were the most popular. The most intriguing finding was that the older population significantly increased their use of all types of telehealth services during the COVID-19 pandemic, and in most types, this use decreased after the COVID-19 peak, but to a level that was higher than the baseline level before the COVID-19 pandemic. Before the COVID-19 pandemic, 23.1% (142,936/618,850) of the study population used working-hours telehealth visits, and 2.2% (13,837/618,850) used after-hours consultations at least once. The percentage of use for these services increased during the COVID-19 pandemic to 59.2% (366,566/618,850) and 5% (30,777/618,850) and then decreased during the third period to 39.5% (244,572/618,850) and 2.4% (14,584/618,850), respectively (*P*<.001). Multiple patient variables have been found to be associated with the use of the different telehealth services in each period.

**Conclusions:**

Despite the limitations and obstacles, the older population uses telehealth services and can increase their use when they are needed. These people can learn how to use digital health services effectively, and they should be given the opportunity to do so by creating suitable and straightforward telehealth solutions tailored to this population and enhancing their usability.

## Introduction

### Background

Telehealth is the practice of providing patients with long-distance clinical health care through various communication technologies (television, email, telephone, videoconferencing, internet, and radio) when a patient and physician cannot be present simultaneously [1,2]. Telehealth, a more general term, encompasses health-related education programs such as diabetes management and nutrition seminars. It is distinct from telemedicine, which is more particularly concerned with the delivery of clinical care via the internet [3]. Telemedicine, which uses current information and communication technology, blends patient requirements with technological progress, going beyond the boundaries of traditional health care systems [4].

Telecare combines professional remote health care services with technological tools and assistive technologies. It offers a range of services, including training, monitoring, consultation, communication, and consultation to preserve users’ autonomy and improve their quality of life. It is particularly valuable for those who live in remote areas, groups considered vulnerable, and aging populations [1,5].

The world’s population is aging quickly, especially in Europe, according to demographic statistics. Aged populations are now more prevalent than ever in many countries, particularly in the more high-income areas. The percentage of people aged >60 years in the world will double between 2000 and 2050, from approximately 11% to 22%. It is predicted that between 2000 and 2050, the number of persons aged >80 years will double [6]. The aging of the population has resulted in many older people living alone in our communities. Because of instances such as the death of a spouse, older people are increasingly being compelled to singularize or live in a single home [4].

Telecare is used more frequently to assist older people in maintaining their independence and carrying on with their current way of life as the population ages and the prevalence of long-term conditions rises. It appears to be one of the most effective strategies for promoting independent living in a community-dwelling setting because it gives an older person a sense of security and comfort [1,7].

Older people have emerged as one of the primary target demographics for telecare technology in recent years, with a variety of gadgets available for those with long-term medical illnesses as well as for those who have limited mobility or memory issues associated with aging [4,8]. Living at home is associated with a superior quality of life, dignity, and independence, and there is a growing trend among older people to do so rather than age in a health care facility [9,10].

A sizeable portion of the population of older adults have at least 1 chronic illness that necessitates routine monitoring and some level of self-management [11]. However, older patients are less likely to notice signs of an exacerbation before being admitted to the hospital, have less awareness of their disease, and engage in fewer self-management activities [12]. The issue may be resolved by evolving technologies that can notify patients to monitor health status information that can help with at-home self-management [7,13,14]. Although there has been general success for many of the telehealth systems already in use, these technologies are sometimes created without considering how easy they will be for patients and caregivers to use. Although telemedicine offers a way to deliver equitable health care, many people with disabilities find it difficult and challenging to access and use telehealth services [15].

Patients participating in video visits must have the technical knowledge and aptitude to connect to the internet, use and troubleshoot audio-visual equipment, and converse without in-person cues. Due to their limitations or lack of technological skills, many older people might be unable to perform this. In addition, older individuals frequently resist using new technology, especially when it comes to gaining knowledge and learning the skills required to operate computers and other electronic devices [4,16]. Older people may also have changes in their eyesight, hearing, and dexterity in addition to the symptoms of chronic illnesses, which could make it difficult for them to use different telehealth devices [17,18].

Although phone consultations are not ideal for care that necessitates visual assessment, they may increase access for the estimated 6.3 million older people who are unfamiliar with technology or have vision impairment [19]. To safeguard both patients and medical staff during the COVID-19 pandemic, there has been a substantial shift to telemedicine, with video visits being encouraged to see patients at home [3,11].

Telehealth, which allows patients to consult with medical professionals in real time and receive advice on their health issues, has become a basic requirement for the public, especially for those who are in quarantine. Telehealth was the most often used method of service delivery during the pandemic, according to a recent report from the World Health Organization [20]. The study also revealed a trend of rise in telehealth uses as income levels rise; even low-income nations, where 42% of people experienced service interruptions during the COVID-19 pandemic, reported using this technology.

The prevalence of telemedicine unreadiness among Medicare beneficiaries aged ≥65 years in the United States during the COVID-19 pandemic was studied in cross-sectional research in community-dwelling individuals and reported by Lam et al [16]. Patients who met the criteria for unreadiness included those who were older, male, single, Black, or Hispanic; lived in a nonmetropolitan area; and had less education, less income, and worse self-reported health. In total, 72%) of adults aged> 85 years met those criteria.

Despite the difficulties with using technology mentioned earlier, there is a misperception about older adults that they either lack interest in using technology or are unable to use technological platforms. Contrary to that belief, most older persons (70%) own and regularly use a computer, smartphone, or tablet with an internet connection at home [21]. However, just a small percentage of older people are comfortable using telehealth (11%) [21].

### Objectives

Considering the growing phenomena of our aging society and the need to implement telecare for this age group, specifically, this study aimed to explore the use and adoption of 3 types of telehealth services among the older population in Israel before, during, and after the COVID-19 pandemic. Using a quantitative approach, the data have been extracted before the COVID-19 pandemic, throughout the pandemic, and during the months after the peak of the epidemic in Israel.

In addition, we wanted to determine whether the COVID-19 pandemic had an impact on how older adults used telehealth services and, if so, whether those changes would last once the pandemic concluded. The results of this study will enable us to emphasize to health care decision makers the necessity for tailored telemedicine-based care that considers the needs, abilities, and preferences of the older population and adapts over time as those needs change.

## Methods

### Study Population

Clalit Health Services, the largest integrated health care service provider and payer system in Israel, has >4 million active members. Clalit Health Services has a comprehensive health care data warehouse, which integrates hospital and community medical records, laboratory and imaging information, pharmaceutical records, health care expenses, and Ministry of the Interior vital statistics of all the members. Clalit Health Services experiences membership turnover of <1% annually, making it easier to track population trends over time. The inclusion and exclusion criteria are presented in Textbox 1.

**Inclusion and exclusion criteria.**

**Inclusion criteria**

- Membership in Clalit Health Services for at least a year
- Aged ≥65 years at each period

**Exclusion criteria**

- No Clalit Health Services membership
- Aged <65 years at each period

### Ethics Approval

The study was ethically approved by the boards of the Clalit Health Services on January 18, 2021 (reference numbers 826 and COM-0113-21).

### Study Design

This study is a repeated cross-sectional analysis. This type of study looks at data collected at a single point in time, rather than over a period, which is useful for comparing and analyzing the effect of different factors on one another or describing a sample.

### Data Extraction

We explored the telehealth use characteristics of older adults (aged ≥65 years) belonging to Clalit Health Services in several aspects:

1. The use of digital services for administrative tasks such as web-based medical appointment scheduling and physician request submission. This category of eHealth services was named *administrative*.
2. The use of synchronous web-based, telephone, or digital visits with the patient’s personal physician during clinic business hours (through video or telephone), initiated by the patients themselves. This type of consultation was named *working hours telehealth consultations*.
3. The use of web-based or telephone consultations (not with the personal physician) during the evenings, nights, and weekends when the clinics are closed, including the use of a phone, video camera, or “tytocare” equipment [22]. The term *after-hours consultations* was used to describe this form of consultation.

### Data Extraction Periods

Three separate periods were used to collect and analyze the data:

1. Baseline—from February 1, 2019, to the end of February 2020, the period before the COVID-19 pandemic in Israel, which was labeled period 1 (*before*).
2. Initiation—from March 1, 2020, to March 31, 2021, when lockdowns were implemented during the COVID-19 pandemic in Israel. Period 2 (*during*) was used to designate this time frame.
3. Follow-up—from April 1, 2021, to the end of October 2021, following the COVID-19 pandemic peak in Israel. Period 3 (*after*) was used to designate this time frame.

### Study Timeline

**Figure 1:**
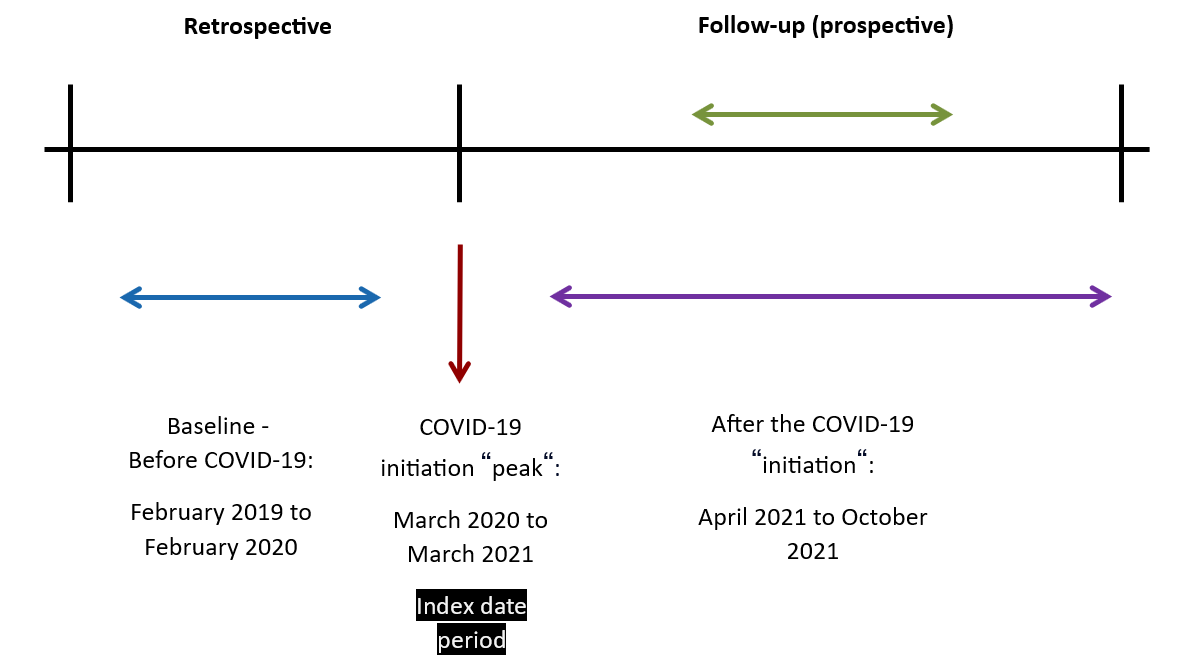
Study design timeline.

Figure 1 describes the timing scheme. The “index date period” refers to the time from March 1, 2020, to March 31, 2021, which is considered the peak of COVID-19 pandemic in Israel. The study was designed and planned during this period.

We planned to gather certain data retrospectively from February 1, 2019, to the index date period and the rest of the data prospectively from the index date period to the end of the follow-up period date, which was October 31, 2021. As intended, and previously indicated, the participant files were recruited from February 1, 2019, to October 31, 2021.

Finally, after gaining approval from all committees of Clalit Health Services, the raw data were accessed for research purposes between February 20, 2022, and May 16, 2022.

### Study Outcomes

As previously indicated, the main outcomes (dependent variables) measured were as follows: (1) The use of digital services for *administrative* tasks, such as web-based medical appointment scheduling and physician request submission, named *administrative services* (2) The use of video or telephone calls with a personal physician conducted during clinic business hours at the patients’ request, named *working-hours telehealth consultations* (3) The use of web-based consultations (not with the personal physician) during the evenings, nights, and weekends when the clinics are closed, including the use of the telephone, video camera, or Tytocare device, named *after-hours consultations*

### Covariables (Covariates)

The inpatient and outpatient data warehouses of Clalit Health Services have provided the data. The information covered the use of telemedicine and digital health services during the aforementioned 3 periods. The variables can be described as dichotomous (use of telemedicine and digital health; yes or no) and as categorical in the aspect of which digital services were used: (1) administrative only use; (2) consultations with the personal physician—*working-hours telehealth consultations*; (3) consultations during the after hours of the clinic—*after-hours consultations.*

Multiple patient variables, such as sociodemographic information (age, sex, place of birth, place of residence, socioeconomic status [SES], ethnicity, country of birth, etc), clinical information (chronic diseases, habits, etc), and use of chronic medications have been examined for each period.

The different features were extracted and categorized as follows: (1) sociodemographic parameters, including sex, age, SES, country of birth (coalesced into regions when necessary), ethnicity by country of individual’s or parents’ birth, sector (clinic level data—predominantly Arab or Jewish), marital status and number of children, Clalit Health Services affiliation by district, subdistrict, and clinic; (2) clinical markers or comorbidities, such as smoking status, alcohol use (and related diagnoses), BMI, height, weight, Charlson comorbidity index (the most widely used comorbidity index used to determine survival rate [1 year and 10 year] in patients with multiple comorbidities), presence of chronic diseases such as active malignancy, cardiovascular diseases (ischemic heart disease, cerebrovascular disease, hypertension), asthma, diabetes, neurological diseases (Alzheimer disease and Parkinson disease), psychiatric disease, diagnosis of COVID-19 since the index period; (3) use of chronic medications, especially antianxiety, antidepression, and sleep-aid medications.

### Statistical Analysis

We analyzed the data according to the type of telehealth use (working hours telehealth consultations, after-hours consultations, and administrative), the time frame in which it was used (before, during, or after the COVID-19 pandemic in Israel), and the amount of use (how often it was used) according to 2 categories (0 or ≥1 time).

We used appropriate descriptive statistics to characterize the study population. The association between telemedicine use and each available socioeconomic factor was studied using univariable analyses while comparing participants who used telemedicine at least once during the specific period with patients with zero use (Pearson *χ*^2^ test). We have used nonparametric related samples Cochran *Q* test to compare telemedicine use during the 3 periods.

Post hoc analysis with the Dunn and McNemar post hoc tests was conducted (with a Bonferroni correction applied) to access both between-subjects and within-subjects effects, analyzing each pair of periods. In addition, we performed a set of multivariable binary logistic regression models to estimate the association between telemedicine use and socioeconomic factors during each period. These models were used to calculate odd ratios (ORs) and 95% CIs. A *P* value <.05 was considered statistically significant. SPSS IBM Statistics for Windows, predictive analytics software (version 28.0 [28.0.1]), was used for data analysis.

### Privacy

Data extraction was conducted by the research room team at Clalit Health Services. The deidentified, raw extracted data were stored on the virtualization desktop infrastructure (VDI), a secure setting. Data were analyzed on the VDI, and only aggregated nonidentifiable results were moved out of the VDI for publication.

As discussed earlier, we did not have any access to identified information. We had limited and restricted access only to unidentified data. A confirmation from the special committee for data mining from Clalit Health Services authorities was received on August 25, 2021.

## Results

### General Characteristics

There were 669,349 patients in total who met the inclusion criteria at the start of period 1 (2019). In 2020, a total of 642,223 patients met the criterion for inclusion, whereas in 2021, only 618,850 patients met the requirements. Various analyses were carried out on this population. The general characteristics are presented in Table 1.

The mean Charlson score was 5.59 (SD 2.57), with a median of 5.00 (range 2-22). The mean age in 2019 was 75.16 (SD 7.64) years, with a median of 73.00 (range 65-110) years.

**Table 1:** General characteristics (N=618,850).

Characteristics		Patients, n (%)
**Sex**		
	Female	349,069 (56.4)
	Male	269,781 (46.6)
**Age group in 2019 (years)**		
	65-74	349,477 (56.5)
	75-84	188,110 (30.4)
	≥85	81,263 (13.1)
**Country of birth**		
	Israel	231,207 (37.4)
	Other	387,643 (62.6)
**Socioeconomic status**		
	Low	146,469 (23.7)
	Medium	235,231 (38)
	High	237,150 (38.3)
**Demographic sector**		
	General Jewish	527,349 (85.2)
	Cherkess	355 (0.1)
	Religious Jewish	12,134 (2)
	Arab	56,692 (9.2)
	Unknown	22,320 (3.6)
**District**		
	South	64,985 (10.5)
	Center	291,145 (47)
	North	168,783 (27.3)
	Center east	93,937 (15.2)
**Smoking status**		
	Never smoked	391,918 (63.3)
	Past smoker	126,853 (20.5)
	Current smoker	61,275 (9.9)
	Status unknown	38,804 (6.3)
**Any chronic disease**		
	Yes	520,771 (84.2)
	No	98,079 (15.8)
**Active malignancy**		
	Yes	57,452 (9.3)
	No	561,398 (90.7)
**Cardiovascular disease**		
	Yes	479,758 (77.5)
	No	139,092 (22.5)
**Asthma**		
	Yes	109,553 (17.7)
	No	509,297 (82.3)
**Diabetes**		
	Yes	237,968 (38.5)
	No	380,882 (61.5)
**Neurological disease**		
	Yes	37,814 (6.1)
	No	581,036 (93.9)

### Telehealth Use Characteristics

#### Overview

Table 2 lists the visit counts (per person) according to the telehealth use type (administrative, working hours, or after hours) and time frame. For telehealth administrative purposes, we can observe that the mean count (per person) was 4.39 (SD 5.56) before the COVID-19 pandemic (period 1), increased to 5.55 (SD 6.71) during the COVID-19 pandemic (period 2), and decreased to 3.07 (SD 4.11) after the peak of the COVID-19 pandemic in Israel (period 3).

Regarding working-hours telehealth consultations with the personal physician, the mean number (per person) was 0.49 (SD 1.43) before the COVID-19 pandemic (period 1), it increased dramatically to a mean of 2.23 (3.54) during the COVID-19 pandemic (period 2), and then it decreased to a mean of 1.00 (1.95) after the peak of the COVID-19 pandemic in Israel (period 3), but still higher than that in period 1.

The mean after-hours telehealth use (per person) was 0.03 (SD 0.02) before the COVID-19 pandemic, increased to 0.07 (0.46) during the COVID-19 pandemic, and decreased again after the peak of the COVID-19 pandemic to 0.03 (SD 0.33).

The visit counts were also analyzed in a dichotomic manner into 2 categories: “no” or “yes,” that is, 0 visits versus ≥1 visit, respectively.

**Figure 2:**
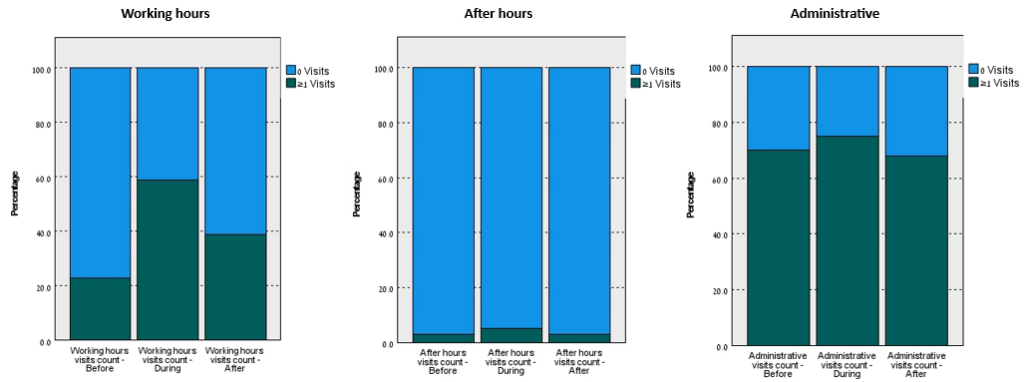
Percentage of Telehealth Services Usage During 3 Periods.

The comparison of telemedicine use (at least once) during 3 periods and the results of Cochran Q test are presented in Figure 2 and Table 3, respectively. The pairwise comparisons of telemedicine use (at least once) during the 3 periods, results of the Dunn and McNemar post hoc tests (with Bonferroni correction), between and within subjects, respectively, are presented in Tables 4 and 5.

**Table 2:** Telehealth use (visit counts) according to types and periods.

Telehealth use		Visits, mean (SD)	Visits, median (IQR)
**Period** **1 (before)**			
	Administrative	4.39 (5.56)	3.00 (0-191)
	Working hours	0.49 (1.43)	0.00 (0-66)
	After hours	0.03 (0.02)	0.00 (0-83)
**Period 2 (during)**			
	Administrative	5.55 (6.71)	4.00 (0-208)
	Working hours	2.23 (3.54)	1.00 (0-104)
	After hours	0.07 (0.46)	0.00 (0-96)
**Period 3 (after)**			
	Administrative	3.07 (4.11)	2.00 (0-107)
	Working hour*s*	1.00 (1.95)	0.00 (0-50)
	After hours	0.03 (0.33)	0.00 (0-96)

**Table 3:** Comparison of telemedicine use (at least once) during 3 periods, results of the Cochran Q test (N=618,850).

Period	Working-hours telehealth visits, n (%)^a^	After-hours visits, n (%)^a^	Administrative, n (%)^a^
Before	142,936 (23.1)	13,837 (2.2)	427,295 (69)
During	366,566 (59.2)	30,777 (5)	459,622 (74.3)
After	244,572 (39.5)	14,584 (2.4)	420,209 (67.9)

^a^*P*<.001.

**Table 4:** Pairwise comparison of telemedicine use (at least once) during 3 periods and results of the Dunn post hoc test (between-subjects analysis with Bonferroni correction).

Sample 1/sample 2	Working-hours telehealth visits			After-hours visits			Administrative	
	Test statistic	Adjusted *P* value	Test statistic		Adjusted *P* value	Test statistic		Adjusted *P* value
Before/after	−0.164	<.001	0.001		1.000	−0.011		.975
Before/during	−0.361	<.001	0.027		<.001	0.052		<.001
After/during	0.197	<.001	−0.026		<.001	−0.064		<.001

**Table 5:** Pairwise comparison of telemedicine use (at least once) during the 3 periods and results of the McNemar post hoc test (within-subjects analysis with Bonferroni correction).

Sample 1/sample 2	Working-hours telehealth visits			After-hours visits			Administrative	
	Chi-square^a^ (*df*)	*P* value	Chi-square (*df*)		*P* value	Chi-square (*df*)		*P* value
Before/after	49,365 (1)	<.001	22 (1)		<.001	462 (1)		<.001
Before/during	184,875 (1)	<.001	7407 (1)		<.001	11,593 (1)		<.001
After/during	72,425 (1)	<.001	6702 (1)		<.001	16,358 (1)		<.001

^a^Continuity corrected.

#### Working-Hours Telehealth Visits (During the Regular Work Hours, With the Personal Physician)

During the first period, 23.1% (142,936/618,850) of the study population used working-hours telehealth services at least once. The percentage of use increased to 59.2% (366,566/618,850) during the second period and then decreased to 39.5% (244,572/618,850) during the third period (still higher than that in period 1).

Pairwise comparisons were performed using the Dunn [23] procedure (between-subjects analysis) with a Bonferroni correction for multiple comparisons. Adjusted *P* values are presented in Table 4. Compared to the percentage of working-hours telehealth services use during the first period, there was a statistically significant increase in the percentage of use during the second period (*P*<.001) and during the third period (*P*<.001). There was also a statistically significant decrease in the percentage of the study population that used working-hours telehealth services at least once during the third period compared to the second period (*P*<.001).

Similar results were demonstrated using the McNemar post hoc test (within-subjects analysis with Bonferroni correction), as demonstrated in Table 5.

#### After-Hours Telemedicine

During the first period, 2.2% (13,837/618,850) of the study population used after-hours telemedicine services at least once. The percentage of use increased to 5.0% (30,777/618,850) during the second period and then decreased to 2.4% (14,584/618,850) during the third period (still higher than that in period 1).

Pairwise comparisons were performed using the Dunn [23] procedure (between-subjects analysis) with a Bonferroni correction for multiple comparisons. Adjusted *P* values are presented in Table 4. Compared to the percentage of after-hours services use during the first period, there was a statistically significant increase in the percentage of use during the second period (*P*<.001), while there was no statistically significant difference compared to the third period. There was also a statistically significant decrease in the percentage of the study population that used after-hours telemedicine services at least once during the third period compared to the second period (*P*<.001).

When using the McNemar post hoc test (within-subjects analysis with Bonferroni correction), significant differences were demonstrated between all 3 pairs of periods, as demonstrated in Table 5.

#### Administrative Telemedicine

During the first period, 69% (427,295/618,850) of the study population used administrative telemedicine services at least once. The percentage of use increased to 74.3% (459,622/618,850) during the second period and then decreased to 67.9% (420,209/618,850) during the third period. Pairwise comparisons were performed using the Dunn [23] procedure (between-subjects analysis) with a Bonferroni correction for multiple comparisons. Adjusted *P* values are presented in Table 4. Compared to the percentage of administrative services use during the first period, there was a statistically significant increase in the percentage of use during the second period (*P*<.001), while there was no statistically significant difference compared to the third period. There was also a statistically significant decrease in the percentage of the study population that used administrative telemedicine services at least once during the third period compared to the second period (*P*<.001).

When using the McNemar post hoc test (within-subjects analysis with Bonferroni correction), significant differences were demonstrated between all 3 pairs of periods, as demonstrated in Table 5.

### Univariate Analysis of Working-Hours Telehealth Visits

According to the dichotomous classification into 2 categories (0 uses versus ≥1 uses), a univariate analysis was conducted using Pearson *χ*^2^ tests on the various types of telehealth use, and the results are shown in Multimedia Appendices 1-3. Multimedia Appendix 1 demonstrates the significant factors associated with the working-hours telehealth visits (telehealth services with the personal physician), with the 2 categories classification (0 visits vs ≥1 visits).

#### Gender

A higher percentage of female individuals than male individuals used the web-based visits at all periods. In addition, both male individuals and female individuals increased their working-hours telecare use during the COVID-19 pandemic period, which was followed by a decline, though at a higher level than before (period 1).

#### Age Group

The age group of 75 to 84 years had used the web-based services to a greater extent, compared to the other age groups (65-74 years and >85 years), at all the 3 periods. In addition, during the COVID-19 pandemic, all age groups significantly boosted their use of working-hours telehealth services; following the COVID-19 pandemic, the use reduced but remained significantly greater than it was before the COVID-19 pandemic (period 1).

#### Country of Birth

Before the COVID-19 pandemic, persons who were born in Israel used working-hours telehealth services more frequently than those who were born elsewhere. However, this tendency shifted during and after the COVID-19 pandemic, when those born outside of Israel had a larger use. Both groups had increased their use of working-hours telehealth services during the COVID-19 pandemic, and it had declined after that time to a greater level than it had been before the COVID-19 pandemic.

#### SES Level

Persons belonging to a higher SES level had a higher level of working-hours telehealth services use, compared to persons with lower SES, at all periods. Here also, we see that during the COVID-19 pandemic, persons in all SES levels greatly expanded their use of working-hours telehealth services; this use eventually declined, but it remained higher than it had been before the COVID-19 pandemic.

#### Demographic Sector

If we ignore the “unknown” portion (which accounts for only 3.6% of the populations included), we see that the religious Jewish population (followed by the general Jewish population) had higher use of working hours telehealth services, as compared to other sectors, at all periods. Here again, persons in all demographic sectors greatly expanded their use of working hours telehealth services during the COVID-19 pandemic period; this use eventually declined, but it remained higher than it had been before the COVID-19 pandemic.

#### District

People from the northern region of Israel had more working hours telehealth visits than people from other districts before the COVID-19 pandemic (period 1). Those from the center districts, however, made many more working-hours telehealth visits to their physician both during and after the COVID-19 pandemic. As stated in the factors described earlier, all subgroups had increased their use of working-hours telehealth services during the COVID-19 pandemic period, and it had declined after that time to a greater level than it had been before the COVID-19 pandemic.

#### Smoking Status

Those who were past smokers used the working hours telehealth visits more frequently than people who are currently smokers or even people who had never smoked. This held true throughout all periods. All subgroups had increased their use of working-hours telehealth services during the COVID-19 pandemic, and it had decreased after that time to a greater degree than it had been before the COVID-19 pandemic, as reflected in the factors mentioned earlier.

#### The Presence of a Chronic Disease

Across all periods, those with chronic diseases significantly used working-hours telehealth visits to a greater extent (more than twice as often as people without chronic diseases). Both groups had expanded their use of working hours telehealth services during the COVID-19 pandemic, and use declined after that period to a larger extent than it had before (period 1).

### Univariate Analysis of Administrative Telehealth Use

Multimedia Appendix 2 shows a univariate analysis, outlining the important variables that were significantly linked to administrative telehealth use in accordance with the 2 categories used (0 visits vs ≥1 visits).

#### Sex

At all 3 periods, a greater proportion of female individuals than male individuals used the administrative web-based services. In contrast to the situation outlined for web-based visits, the administrative telehealth use (for this parameter and all other parameters described here) did increase throughout the COVID-19 pandemic before declining to a level below that of the before period (period 1).

#### Age Group

The age group of 75 to 84 years had used the administrative web-based services in a greater extent, compared to the other age groups (65-74 years and >85 years), at all the 3 periods. In addition, as reported earlier, during the COVID-19 pandemic, all age groups significantly boosted their use of web-based services, but the use reduced following the COVID-19 pandemic, to a lower level than it was before the COVID-19 pandemic.

#### Country of Birth

Throughout all periods, those born outside of Israel used administrative web-based services more than those who were born there. Both groups had increased their use of web-based services during the COVID-19 pandemic, and it had declined after that time to a lower level than it had been before the COVID-19 pandemic.

#### SES Level

Persons belonging to a higher SES level had a higher level of administrative web-based services use, compared to persons with lower SES, at all periods. Here also, we see that during the COVID-19 pandemic, persons in all SES levels greatly expanded their use of web-based services; this use eventually declined to a lower level than it had been before the COVID-19 pandemic.

#### Demographic Sector

Throughout all periods, the Cherkess (Circassians) had the higher use of administrative web-based services, as compared to other sectors. In this instance, as well, people in all demographic groups significantly increased their use of administrative web-based services during the COVID-19 pandemic; nevertheless, this use gradually decreased to a level that was lower than it had been before the COVID-19 pandemic.

#### District

People from Israel’s southern region used administrative internet services to a greater extent than residents from other districts at all times. All subgroups had increased their use of administrative web-based services during the COVID-19 pandemic, and it had decreased after that time to a lower level than it had been before the COVID-19 pandemic, as was shown in other factors mentioned earlier.

#### Smoking Status

People who had previously smoked used the administrative web-based services more than those who smoke now or even those who have never smoked. This was accurate at all times. All subgroups had grown their use of administrative web-based services during the COVID-19 pandemic and had declined after that period to a lesser extent than it had been before the COVID-19 pandemic.

#### The Presence of a Chronic Disease

Throughout all periods, persons with chronic conditions significantly used the administrative web-based services more frequently (more than twice as often as people without chronic diseases). Both groups had expanded their use of web-based services during the COVID-19 pandemic, and use decreased after that period to a lower extent than it had before (period 1).

As demonstrated in Table 2, the overall use of after-hours telemedicine services (during the evenings, nights, and weekend days) was considerably lower than that of administrative telehealth and working-hours telemedicine services (with the personal physician during opening hours).

### Univariate Analysis of After-Hours Telemedicine Use

In accordance with the 2 categories used (0 visits vs ≥1 visits), Multimedia Appendix 3 presents a univariate analysis summarizing the relevant variables that were significantly associated to after-hours telemedicine use:

#### Sex

At all 3 periods, a greater proportion of females than males used the after-hours telemedicine services. During the COVID-19 pandemic, both men and women increased their use of after-hours telemedicine services, which was followed by a fall, but at a greater level than previously (period 1), in a manner similar to the trend outlined for the working-hours telehealth services.

#### Age Group

Only on the COVID-19 pandemic, the age groups of 65 to 74 years and 75 to 84 years had significantly greater use of after-hours telemedicine services, compared to the third age group (>85 years). In addition, as previously mentioned, during the COVID-19 pandemic, all age groups increased their use of after-hours services; however, after the COVID-19 pandemic, use decreased, though it remained at a higher level than it had been before the COVID-19 pandemic.

#### Country of Birth

During the COVID-19 pandemic and the period after the COVID-19 pandemic, those born outside of Israel significantly used after-hours telemedicine services more than those who were born there. Both groups had increased their use of after-hours telemedicine services during the COVID-19 pandemic, and it had declined after that time (for people born outside Israel, it declined to a higher level than it was before the COVID-19 pandemic).

#### SES Level

Similar to how it was with the other telehealth services, those with higher SES levels used after-hours telemedicine services more frequently than people with lower SES levels did always. In this area as well, the use of after-hours services by people of all SES levels significantly increased during the COVID-19 pandemic. Eventually, this use decreased but it did so at a greater level than it had been before the COVID-19 pandemic.

#### Demographic Sector

As described for the use of working-hours telehealth visits, here also, the religious Jewish population and the general Jewish population had the higher use of after-hours services, as compared to other sectors, at all periods (the religious Jews had a higher use during the COVID-19 pandemic). Here again, people in all demographic sectors greatly expanded their use of after-hours services during the COVID-19 pandemic, which declined after that period.

#### District

Contrary to the previously mentioned telehealth services, persons from Israel’s central area were much more likely to use after-hours telemedicine services than those from other regions during all periods. The use of after-hours internet services had surged across all subgroups during the COVID-19 pandemic and had fallen thereafter to a greater level than it had been before the COVID-19 pandemic (except for the south region in which it declined to the same level as before).

#### Smoking Status

As was the case with the other telehealth services, people who had previously smoked used more the after-hours services than those who do so now or even those who have never smoked. This was accurate at all periods. All groupings had increased their use of after-hours internet services during the COVID-19 pandemic and had decreased after that typically to a greater extent than it had been before the COVID-19 pandemic.

#### The Presence of a Chronic Disease

Similar to other telehealth services mentioned earlier, those with chronic conditions significantly used the after-hours web-based services more often across all periods (more than twice as often as people without chronic diseases). During the COVID-19 pandemic, both groups (with and without chronic diseases) increased their use of web-based services, and after that time, use decreased but to a higher level than it had previously (period 1).

### Multivariate Analysis

In the multivariate analysis, using a set of multivariable binary logistic regression models, several parameters were found to be significantly associated with the different types of telehealth use during each period: male sex (decreased use of all types of telehealth services, during all periods); country of birth–Israel (increased use of working-hours telehealth visits and after-hours visits during all periods but decreased administrative use at all periods); presence of any chronic disease (highly increased use of all types of telehealth services during all periods); Charlson comorbidity score (increased use of all types of telehealth services during all periods); medium and high SES (increased use of all types of telehealth services during all periods); Jewish religion (highly increased use of all types of telehealth services during all periods); southern district—place of residence (people living in this district used fewer working-hours telehealth services and fewer after-hours services during all periods but used more administrative services before and after the COVID-19 pandemic); northern district (people living here used more working-hours telehealth services before the COVID-19 pandemic but used less working-hours telehealth services during periods 2 and 3 and used fewer administrative services and fewer after-hours services at all periods); central east district (people living here used fewer of all types of telehealth services during all periods); current or past smokers (these people used more working-hours telehealth services, more after-hours services, and more administrative services during all periods); older age groups of those aged ≥75 years (used fewer working-hours telehealth services, fewer after-hours services, and fewer administrative services during all periods).

The multivariate analysis is demonstrated in Table 6 (for working-hours telehealth visits), Table 7 (for after-hours visits), and Table 8 (for administrative uses). The Nagelkerke *R*^2^ values for each type of telehealth service and each period are also provided in the tables.

**Table 6:** Multivariate analysis—working-hours telehealth visits. Variables entered on step 1: sex (male), country of birth, any chronic disease, Charlson score, SES (medium), SES (high), Jewish nationality, district (south), district (north), district (central east), current or past smoker, age group (75-84 years), age group (≥85 years).

Working-hours visits	Variables in the equation										
	Before (period 1)^a^				During (period 2)^b^				After (period 3)^c^		
	*P* value	Exp (B)	95% CI	*P* value		Exp (B)	95% CI	*P* value		Exp (B)	95% CI
Sex (male)	<.001	0.717	0.708**-**0.726	<.001		0.741	0.733-0.749	<.001		0.701	0.694-0.709
Country of birth (Israel)	<.001	1.079	1.064**-**1.094	<.001		1.108	1.094-1.121	<.001		1.068	1.055-1.081
Any chronic disease	<.001	1.978	1.934-2.022	<.001		2.612	2.571-2.654	<.001		2.197	2.159-2.237
Charlson score	<.001	1.144	1.141-1.147	<.001		1.201	1.198-1.204	<.001		1.156	1.154-1.159
SES^d^ (medium)	<.001	1.332	1.308**-**1.355	<.001		1.375	1.355-1.395	<.001		1.288	1.269-1.307
SES (high)	<.001	1.374	1.349**-**1.400	<.001		1.587	1.562-1.612	<.001		1.438	1.416-1.461
Jewish religion	<.001	1.065	1.042-1.088	<.001		1.318	1.294-1.343	<.001		1.277	1.253-1.301
District (south)	<.001	0.653	0.638**-**0.668	<.001		0.876	0.860-0.892	<.001		0.806	0.791-0.821
District (north)	<.001	1.109	1.092**-**1.125	<.001		0.816	0.805-0.827	<.001		0.924	0.913-0.937
District (central east)	.01	0.977	0.960**-**0.995	<.001		0.868	0.854-0.882	<.001		0.816	0.803-0.829
Current or past smoker	<.001	1.152	1.136**-**1.168	<.001		1.191	1.176-1.205	<.001		1.095	1.082-1.108
Age group (75-84 years)	<.001	0.818	0.806**-**0.830	<.001		0.922	0.910-0.934	<.001		0.811	0.801-0.821
Age group (≥85 years)	<.001	0.743	0.728**-**0.758	<.001		0.809	0.794-0.824	<.001		0.647	0.635-0.658
Constant	<.001	0.068	N/A^e^	<.001		0.169	N/A	<.001		0.124	N/A

^a^Nagelkerke *R*^2^=0.065.

^b^Nagelkerke *R*^2^=0.139.

^c^Nagelkerke *R*^2^=0.090.

^d^SES: socioeconomic status.

^e^N/A: not applicable.

**Table 7:** Multivariate analysis—after-hours telehealth visits. Variables entered on step 1: sex (male), country of birth, any chronic disease, Charlson score, SES (medium), SES (high), Jewish nationality, district (south), district (north), district (central east), current or past smoker, age group (75-84 years), age group (≥85 years).

After-hours visits	Variables in the equation								
	Before (period 1)^a^			During (period 2)^b^			After (period 3)^c^		
	*P* value	Exp (B)	95% CI	*P* value	Exp (B)	95% CI	*P* value	Exp (B)	95% CI
Sex (male)	<.001	0.748	0.722**-**0.775	<.001	0.850	0.829-0.870	<.001	0.799	0.772-0.827
Country of birth (Israel)	<.001	1.086	1.046**-**1.128	.01	1.034	1.007-1.061	.12	1.030	0.992-1.069
Any chronic disease	<.001	2.063	1.925-2.210	<.001	1.813	1.736-1.893	<.001	1.701	1.598-1.810
Charlson score	<.001	1.111	1.103**-**1.118	<.001	1.091	1.086-1.096	<.001	1.103	1.096-1.111
SES^d^ (medium)	<.001	1.264	1.199**-**1.333	<.001	1.213	1.171-1.257	<.001	1.208	1.148-1.271
SES (high)	<.001	1.404	1.330**-**1.482	<.001	1.330	1.282-1.379	<.001	1.339	1.271-1.411
Jewish religion	<.001	1.981	1.837**-**2.136	<.001	1.679	1.600-1.762	<.001	1.821	1.694-1.956
District (south)	<.001	0.792	0.745**-**0.842	<.001	0.741	0.711-0.773	<.001	0.793	0.747-0.842
District (north)	<.001	0.767	0.734-0.801	<.001	0.646	0.627-0.666	<.001	0.718	0.687-0.749
District (central east)	<.001	0.850	0.809-0.893	<.001	0.838	0.811-0.867	.001	0.923	0.880-0.967
Current or past smoker	.001	1.067	1.028-1.108	.01	1.034	1.007-1.061	.02	1.045	1.008-1.085
Age group (75-84 years)	<.001	0.780	0.749-0.812	<.001	0.807	0.785-0.830	<.001	0.818	0.786-0.851
Age group (≥85 years)	<.001	0.730	0.691**-**0.772	<.001	0.666	0.640-0.693	<.001	0.736	0.697-0.777
Constant	.000	0.004	N/A^e^	<.001	0.013	N/A	<.001	0.005	N/A

^a^Nagelkerke *R*^2^=0.027.

^b^Nagelkerke *R*^2^=0.027.

^c^Nagelkerke *R*^2^=0.022.

^d^SES: socioeconomic status.

^e^N/A: not applicable.

**Table 8:** Multivariate analysis—administrative telehealth visits. Variables entered on step 1: sex (male), country of birth, any chronic disease, Charlson score, SES (medium), SES (high), Jewish nationality, district (south), district (north), district (central east), current or past smoker, age group (75-84 years), age group (≥85 years).

Administrative telehealth visits	Variables in the equation									
	Before (period 1)^a^			During (period 2)^b^				After (period 3)^c^		
	*P* value	Exp (B)	95% CI	*P* value	Exp (B)	95% CI	*P* value		Exp (B)	95% CI
Sex (male)	<.001	0.788	0.778-0.798	<.001	0.790	0.780-0.800	<.001		0.818	0.808-0.827
Country of birth (Israel)	<.001	0.938	0.926-0.951	.34	0.993	0.979-1.007	<.001		0.974	0.962-0.987
Any chronic disease	<.001	3.690	3.631-3.750	<.001	3.947	3.883-4.013	<.001		3.742	3.682-3.803
Charlson score	<.001	1.213	1.209-1.216	<.001	1.237	1.233-1.241	<.001		1.200	1.196-1.203
SES^d^ (medium)	<.001	1.355	1.333-1.377	<.001	1.436	1.412-1.460	<.001		1.352	1.331-1.373
SES (high)	<.001	1.386	1.362-1.410	<.001	1.558	1.531-1.587	<.001		1.498	1.473-1.523
Jewish religion	<.001	2.291	2.247-2.335	<.001	1.943	1.904-1.982	<.001		2.206	2.164-2.248
District (south)	<.001	1.044	1.023-1.067	.43	0.991	0.969-1.014	.002		1.033	1.012-1.054
District (north)	<.001	0.655	0.646-0.665	<.001	0.647	0.638-0.657	<.001		0.661	0.651-0.670
District (central east)	<.001	0.702	0.690-0.714	<.001	0.724	0.711-0.738	<.001		0.760	0.747-0.773
Current or past smoker	<.001	1.311	1.293-1.329	<.001	1.354	1.334-1.374	<.001		1.289	1.272-1.306
Age group (75-84 years)	<.001	0.753	0.743-0.764	<.001	0.698	0.687-0.708	<.001		0.745	0.734-0.755
Age group (≥85 years)	<.001	0.452	0.444-0.461	<.001	0.364	0.356-0.371	<.001		0.434	0.425-0.442
Constant	<.001	0.162	N/A^e^	<.001	0.209	N/A	<.001		0.155	N/A

^a^Nagelkerke *R*^2^=0.198.

^b^Nagelkerke *R*^2^=0.209.

^c^Nagelkerke *R*^2^=0.193.

^d^SES: socioeconomic status.

^e^N/A: not applicable.

## Discussion

### Principal Findings

In this study, we examined the use and uptake of 3 distinct telehealth services among the older population who were members of Clalit Health Services before, during, and after the COVID-19 pandemic in Israel. Data of 618,850 patients who met the inclusion criteria were extracted. Telehealth services used for administrative purposes were the most popular. The older population significantly increased their use of all types of telehealth services during the COVID-19 pandemic, and in most types of services, this use decreased after the COVID-19 peak but to a level that was higher than the baseline level before the COVID-19 pandemic. The three telehealth service types that were investigated in the study were as follows: (1) “working-hours telehealth visits” refers to video or telephone consultations with a personal physician during clinic business hours; (2) “administrative” refers to the use of digital services for administrative tasks (such as scheduling appointments or submitting requests to the physicians on the web); and (3) “after-hours visits” refers to the use of web-based consultations in the evenings, weekend days, and other nonbusiness hours (with other physicians)

A quantitative method was used to extract the data at three different time points: (1) “before” was before the COVID-19 pandemic; (2) “during“ was during the COVID-19 pandemic’s emergence; and (3) “after” was during the months after the peak of the pandemic in Israel.

Our main objectives were to assess how the older adults in Israel used various telehealth services, considering the challenges and difficulties they encountered; to determine what factors were associated with increased or decreased uptake; and to determine whether the COVID-19 period had any impact on use patterns and whether those patterns persisted after the period had ended.

Tables 2 and 3 show that, throughout the study periods, the telehealth services used for administrative purposes were the most popular among the older population. This was followed by “working-hours telehealth visits”—telemedicine consultations with the personal physician—during the regular business hours of clinics. Among the other services mentioned earlier, “after-hours” telemedicine visits came in last.

Another intriguing finding was that during the COVID-19 pandemic, the older population significantly increased their use of all telehealth services, and this use decreased after the peak of the COVID-19 pandemic (period 3), in all types of telehealth uses (Tables 3 and 4). However, concerning the working-hours telehealth visits, and the after-hours visits—this decrease was found to remain significantly higher than the baseline level before the COVID-19 pandemic.

These findings support our assumptions that as expected, the use of all telehealth services was increased during the COVID-19 pandemic. The working-hours telehealth visits, which are the primary telemedicine meetings (with the personal physician), prospered during the COVID-19 pandemic, but even after this period, they remained at a higher level than they had before the COVID-19 pandemic period.

These findings confirm our hypothesis that older adults are interested in and capable of using telehealth services, given the opportunity and accessibility to do so—factors that were noticeably improved during the COVID-19 pandemic—despite their hesitations and barriers and the medical system’s mistaken belief that there is no point in teaching this population how to use digital health services.

In the univariate analysis, after classifying the number of telehealth services used into 2 categories (0 visits vs ≥1 visits), we found several factors that were significantly associated with increased telehealth use among the older individuals (Multimedia Appendices 1-3). Furthermore, we conducted a multivariate analysis**,** using a set of multivariable binary logistic regression models, which revealed that several parameters were significantly associated with the different types of telehealth utilization during each period (Tables 6-8).

Women were found to significantly use more working-hours telehealth visits with the personal physician, more after-hours telehealth visits, and more administrative telehealth services across all periods compared to men. This finding was supported by the multivariate analysis, showing that among men, there was decreased use of all types of telehealth services, during all periods. This finding may be explained by women’s greater general health literacy, eHealth literacy, and health awareness, as previously reported [24-26].

The findings for telehealth use by age groups in the univariant analysis were somewhat unexpected because it would be reasonable to presume that younger age groups (aged 65-74 years) would use telehealth the most. The findings indicate that the older age groups used telehealth services more frequently. Specifically, those aged 75 to 84 years used more working-hours telehealth visits during opening hours, more administrative telehealth use, and more after-hours visits following the COVID-19 pandemic. However, in the multivariate analysis, older age groups (aged ≥75 years) were found to use fewer working-hours telehealth services, fewer after-hours services, and fewer administrative services, during all periods.

These findings are consistent with the general belief that “younger, more highly educated and affluent seniors use technology more readily and across broader platforms than the older old, who as a group tend to be less affluent, less educated, and often have a significantly greater burden of chronic illness and disability,” as reported by Greenwald et al [21]. These authors claimed that younger seniors, who are more physically and psychologically integrated into the technological modern world, may have a more positive attitude toward the benefits of technology than older seniors. In contrast, the use of automated telephone menu systems, medical-related purchases on the internet (such as medical supplies or medications), and telemedicine videoconferencing with health care providers were all found to be more common among older adults than among younger adults in a 2011 study that looked into the type and frequency of technology use for a variety of health care activities [27].

The findings relating to SES in the univariate analysis were as expected: higher SES was significantly associated with increased telehealth use of all services investigated (working-hours telehealth visits, after hours, and administrative use) at all periods. This was also demonstrated in the multivariate analysis, in which people in medium and high SES significantly had increased use of all types of telehealth services during all periods. This could be ascribed to higher levels of education; increased knowledge and awareness of digital health services; increased income enabling the acquisition of computers, smartphones, and digital devices; and increased eHealth literacy linked to higher SES levels. These findings are in line with earlier studies, which found that people with lower median household incomes and less favorable insurance situations used web-based visits less frequently [26]. Another study also found a correlation between declining SES and declining probabilities of using telemedicine during elective surgery visits [28]. According to comparable findings among 16,000 patients with a new cancer diagnosis, those with the greatest SES were more likely to use telemedicine within 30 days of diagnosis [29].

The demographic sector findings in the univariate analysis were also a little surprising: the religious Jews, compared to other groups, had more working-hours telehealth visits with their physician, at all periods, and more after-hours visits (followed by the general Jewish population), before and during the COVID-19 pandemic. However, only in case of the administrative use, the Cherkess (Circassians) had greater use at all periods. These findings are unexpected given that using telehealth solutions in ultraorthodox communities offers several difficulties given their restricted access to virtual communication as well as their reluctance to engage in this novel modality of therapy. For many of these populations, receiving therapy through the web is strange and foreign, and it may be seen as going against their religious principles [30,31]. Nevertheless, in the multivariant analysis, those of the Jewish religion demonstrated highly increased use of all types of telehealth services during all periods.

The findings regarding the district that the older people belong to (where they reside) in the univariate analysis were inconsistent: before the COVID-19 pandemic, more working-hours telehealth visits were observed in the northern district; however, during and after the COVID-19 pandemic, more working-hours telehealth visits were made in the central areas. In addition, during all periods, more after-hours visits were made by residents of central regions. However, throughout all periods, people from the southern district used telehealth more frequently for administrative functions.

Using the multivariate analysis, we saw that people living in southern district used fewer working-hours telehealth services and fewer after-hours services during all periods but used more administrative services before and after the COVID-19 pandemic; people living in the northern district used more working-hours telehealth services before the COVID-19 pandemic but used less working-hours telehealth services during periods 2 and 3 and used fewer administrative services and fewer after-hours services in all periods; and people living in the central east district used less of all types of telehealth services during all periods. In general, we may conclude that a more peripheral place of residency was associated with reduced use of telehealth services. These findings are unexpected because one may anticipate that telehealth services would be used more frequently in remote places, where there are typically fewer health care personnel and resources.

The association of smoking status with telehealth use in the univariate analysis was interesting: for all types of telehealth services (working-hours telehealth visits with the personal physician, after-hours visits, and administrative telehealth use), people who had smoked in the past and stopped smoking (past smokers) substantially used more services compared to other groups (even more than nonusers) at all periods. This may be accounted for by such people’s greater health awareness, which led to a major change in their health-related behaviors. However, the multivariate analysis demonstrated that current or past smokers used more working-hours telehealth services, more after-hours services, and more administrative services during all periods. These findings could be additionally explained by the notion that current smokers have greater health problems, necessitating more frequent appointments with health care providers.

Finally, compared to persons without chronic diseases, people with chronic diseases significantly used digital services more frequently for administrative tasks, after-hours telehealth visits, and working-hours telehealth visits during business hours. This was true throughout all periods (including those before, during, and after the COVID-19 pandemic). This group’s rising use of telehealth was more than twice as high as that of people who were ordinarily healthy. These findings were also supported by the multivariate analysis demonstrating that the presence of any chronic disease was significantly associated with highly increased use of all types of telehealth services during all periods; in addition, the Charlson comorbidity score was found to be significantly associated with increased use of all types of telehealth services during all periods. This is not unexpected given that individuals with chronic illnesses or comorbid conditions may require additional help from health care professionals, and they frequently experience accessibility issues, making telehealth services particularly desirable to them.

In this study, we investigated the use of telehealth by older people who are still living at home to communicate with their clinic (administrative requests); individual physicians; or other medical professionals on evenings, weekend days, and holidays when clinics are closed. Nevertheless, there are telecare options available globally that enable the monitoring of patients with a chronic illness, such as heart failure, hypertension, diabetes, asthma, chronic obstructive pulmonary disease, or stroke [32-34]. In addition, more health care systems are using telemedicine video communication as a tool for health maintenance after discharge to lower hospital readmissions as well as expedited consult services (stroke, trauma, mental health screening, and surgical second opinions) [21]. Evaluation of telehealth programs for individuals, particularly the older adults, with chronic medical issues has yielded conflicting outcomes. Glycemic management and the use of health care services both benefited patients with diabetes [35].

The research on telemedicine-enhanced emergency care for older people has been concentrated on residents of older people living community centers and has shown that high-intensity telemedicine services for acute illnesses have been effectively carried out, believed to be acceptable by older patients, and offered definitive care without needing a referral to the emergency department or urgent care [36]. An analysis of the impact of home-based telehealth interventions on the use of secondary care and mortality in a cohort of patients with COPD (chronic obstructive pulmonary disease), diabetes, and heart failure, most of whom (70%) were aged >65 years, led to lower emergency admission rates and lower mortality. [37] However, a different analysis of the same data revealed no impact on psychological outcomes or quality of life over a 12-month period [38].

### Benefits of Telemedicine

It is well agreed that telemedicine-based care offers many benefits and advantages for older people. Older adults who use eHealth services can maintain their freedom and continue to live in their own familiar homes, where they feel secure and at ease. Their sense of security and quality of life are improved by being aware that they are constantly being watched and monitored [39-41]. According to Chou et al [40], older people who frequently used their telecare program and had better social welfare and health status also embraced using technology and had a higher quality of life. Their findings also showed that older people who believed telecare could help them with their health issues and were prepared to use it had a higher opinion of their quality of life, particularly in terms of their social interactions and home environment.

When an older person has decreased mobility, easy and quick web-based communication with a health care facility or professional becomes particularly crucial. This reduces travel time, speeds up diagnosis, reduces the need for repeat diagnostic tests and clinical services, and allows for older adult triage that is appropriate [42]. By offering some medical services at the patient’s home, telemonitoring and telerehabilitation help to decrease the frequency of hospitalizations and shorten their length, and the patient who is chronically ill can benefit by reducing the number of follow-up visits required at the medical facility [42-44]. In addition, due to telecare systems using eHealth services, an older person who is housebound can sign up via the web for a physician’s appointment, choose to receive a reminder of a due date for a medical consultation, receive straightforward remote medical recommendations or test results (via SMS text messaging or email), and buy ongoing prescription drugs from the pharmacy of their choice [45,46].

By allowing patients to develop and select the tools they will use as well as how they will use them, telecare fosters increased involvement [47]. Patients can actively participate in their own care through the use of telecare systems rather than just receiving it as a passive benefit. They become partners on an equal footing with their physicians, capable of making choices for their health on their own while being cognizant of the repercussions [48,49].

Through telemedicine, proactive healthy behaviors are formed. During an emergency, telemonitoring can identify pathological signs and symptoms and abnormal test results earlier than during or before a typical physician’s visit or examination, enabling prompt preventive action [42]. Telemonitoring also has a substantial impact on education. Patients who are conscious of their health status frequently begin to educate themselves on their disease and how to self-manage it. They have a better understanding of their body and are more motivated to modify unhealthy behaviors and live healthier lifestyles [42,50].

Telecare lessens socioeconomic and regional inequities in access to care as well as the uneven distribution of care quality. With the help of telecare technologies, older people can easily connect with a variety of functionally and geographically dispersed health care professionals at times that are convenient for both the patient and the health care provider [42].

Physicians in varied practice settings can easily access evidence-based medicine and effective clinical decision-making tools, such as knowledgeable colleagues in tertiary care facilities. In addition, patients can get the right type of care close to where they live, which is crucial for older people with chronic illnesses or disabilities because it affects their quality of life and level of care [42,51]. According to Chae et al [52], telecare was successful in lowering the frequency of clinic visits and also increased patient satisfaction in a trial of home health services for older people.

### Barriers to Telecare Use

Although telecare offers undeniable advantages, it is important to understand that it also has limits, some of which are due to the older people themselves. The use of new technologies is frequently resisted by older individuals. Although computers and the internet have become important tools, older adults experience more trouble using them than younger people do.

According to a study by the Nielsen Norman Group, users aged >65 years had a success rate of just 53% when completing a series of assigned tasks (such as finding information and making a web-based purchase), compared to a group of younger users who had a success rate of 78%. In addition, the older group made an average of 3.7 errors on each task given, as opposed to the younger group’s average of 0.6 errors [53]. Another issue is the decline in cognitive and motor function that comes with aging (eg, vision, hearing, short-term memory loss, and physical impairment), which makes it harder to adapt to a changing environment and assimilate new behavioral patterns [54].

The strong desire to interact directly and personally with the physician is another trait shared by older people. They typically prefer face-to-face interactions with health care providers, so telemedicine-based services delivered remotely are frequently not seen as relevant to them. In a study on older adults aged ≥60 years, Bujnowska-Fedak and Mastalerz-Migas [55] found that 61% of older adults stated a strong preference for direct contact with medical professionals as the main deterrent to contacting their family physician, specialist, or nurses via telephone or the web. Eliminating in-person care may give older persons the impression that they are engaging in less social interactions. Resistance to telemedicine in older population may be a result of their concern that the new technology will negatively impact their social and personal relationships [56].

The next barrier is money. Pensioners, typically those who are in need, worry about the high prices of buying computers or other electronics. Older persons are frequently reluctant to spend money on home health care monitoring systems, despite the health advantages and long-term cost reductions made available by telecare services [55]. In addition, for older persons, privacy and security are top personal concerns. They want more assurance that their private information is kept private from prying eyes. Better health care is not as important to them as feeling assured about the security of their medical information [56,57].

### Accelerating Growth of Care Based On Telemedicine

Technology advancements have made it feasible to put into practice solutions that, up until recently, looked to be a long way off. Israel is regarded as a highly developed country with excellent infrastructure, a high degree of entrepreneurship and innovation, and widespread knowledge of telemedicine services. As part of the Digital Israel Project, the Ministry of Health declared that one of its objectives was to “bring about a leap in the health system that will enable it to become sustainable, advanced, innovative, renewed, and constantly improving, by optimally leveraging the information and communication technologies available to the entire Israeli population” [58]. Similar to other developed nations, Israel has seen a rise in telemedicine use because of the COVID-19 pandemic.

Telecare improves the quality of life of older citizens with chronic illnesses worldwide by providing them with new options for education, prevention, diagnosis, treatment, and rehabilitation. It equalizes possibilities for patients from urban and rural locations and lessens socioeconomic gaps in access to care. According to a prior study [45], 41% of older people had a favorable opinion of eHealth services and were willing to use them if and when given the chance. A considerable shift from passive monitoring to more active use of telecare technologies that enable and promote direct connection has occurred in well-developed countries in recent years. Patients now have more control over their own health and welfare because of the changing health care system [43]. They can make health-related decisions on their own and with knowledge of the repercussions, and they work as equal partners with their physicians [59,60].

Nevertheless, widespread acceptance by older persons themselves is a crucial component in the development of telecare systems for the older population [43,51]. Despite the growing popularity of computers and mobile devices among older people and improved computer literacy, many of them are still unaware of the opportunities that telemedicine presents. Training for the use of telecare appears to be quite vital, as does ongoing education of the older population in this area. The needs, abilities, and preferences of older people should now be taken into account while providing telemedicine-based care, with adjustments made over time as care requirements change. Older adults have a wide range of needs, which can alter with time. Therefore, it is essential to individualize and adapt telecare systems for a range of abilities of older people, addressing their changing care requirements in a flexible and adaptable manner, always considering their impaired motor, sensory, and cognitive function.

Before using telecare technologies, all older people must be familiar with their utilization and aware of their advantages. In addition, perceptions of older people and their caregivers about the usability of home telecare are a substantial predictor of compliance with telecare [51]. An equitable health system should understand that while many older persons are willing and able to learn how to use telemedicine, for some, such as those with dementia and social isolation, in-person visits are already challenging, and telemedicine may be impractical. Clinics and geriatric modes of treatment, such as home visits, are crucial for these individuals [59].

Telecare will soon become a crucial aspect of older people’s lives, enabling them to function independently in a comfortable living environment, if technologies are developed that are familiar, usable, appealing, affordable, and fit into lives and plans of older people. Further research is required to accentuate the importance of offering the older population telehealth alternatives that are both easily accessible and easy to use.

### Limitations

Despite the fact that this study included a large set of data from people belonging to Israel’s largest health management organization (and one of the largest health care organizations in the world), it still represents trends in telehealth use among the older population in Israel and not necessarily in all parts of the world. Furthermore, there were several parameters that could have an additional impact on telehealth use (eg, level of education); however, we did not have access to these data.

### Conclusions

It is generally acknowledged that telemedicine-based treatment for the older people has several advantages. However, telemedicine also has limitations and barriers, some of which are due to the older people themselves.

The key findings of our study demonstrate that, despite all the challenges and hurdles, the older population uses telehealth services when they need them. People use telehealth services for administrative purposes more frequently, but they also consult with their own physician via telephone and the web and sometimes even use after-hours virtual consultations. These services make it easier for individuals to get medical care without having to travel, wait, or risk infection. Older people can increase their consumption as necessary during times of pressing necessity, such as the COVID-19 pandemic, or if they are afflicted with a persistent illness.

The study also reveals that even after the COVID-19 pandemic, most uses remained higher than they were before, implying that this population can learn how to use digital health services effectively and that they should be given the opportunity to do so by creating suitable and straightforward telehealth solutions tailored for this population and enhancing their usability.

## Appendix

Multimedia Appendix 1 Univariate analysis (working-hours visits)—2 categories.

Multimedia Appendix 2 Univariate analysis (administrative telehealth use)—2 categories.

Multimedia Appendix 3 Univariate analysis (after hours visits)—2 categories.

## Abbreviations

COPD: chronic obstructive pulmonary disease
OR: odds ratio
SES: socioeconomic status
VDI: virtualization desktop infrastructure

## Data Availability

All data generated or analyzed during this study are included in this published article and its supplementary information files.
