# Supplementary material for: Adoption and Use of Telemedicine and Digital Health Services Among Older Adults in Light of the COVID-19 Pandemic: Repeated Cross-Sectional Analysis
Source: JMIR Aging. 2024 Apr 24;7:e52317. doi: 10.2196/52317 (PMC11079757; doi:10.2196/52317)
Supplement: Multimedia Appendix 1 [file aging_v7i1e52317_app1.docx]

**Appendix 1:** **Univariate analysis- Working-hours visits – 2 categories**

|  | | **Working-hours visits telehealth Count - Before (0/1+)** | | | **Working-hours telehealth visits Count - During (0/1+)** | | | **Working-hours telehealth visits Count - After (0/1+)** | | |
| --- | --- | --- | --- | --- | --- | --- | --- | --- | --- | --- |
|  |  | **0 Visits** | **1+ Visits** | **P- value** | **0 Visits** | **1+ Visits** | **P- value** | **0 Visits** | **1+ Visits** | **P- value** |
|  |  | **%** | **%** |  | **%** | **%** |  | **%** | **%** |  |
| **Sex (male)** | **Total** | 76.9% | 23.1% | P=.000* | 40.8% | 59.2% | P=.000* | 60.5% | 39.5% | P=.000* |
|  | **FEMALE** | 75.4% | 24.6% |  | 39.3% | 60.7% |  | 58.2% | 41.8% |  |
|  | **MALE** | 78.9% | 21.1% |  | 42.7% | 57.3% |  | 63.4% | 36.6% |  |
| **Age group in 2019** | **65-74** | 77.5% | 22.5% | P=.000* | 43.6% | 56.4% | P=.000* | 61.2% | 38.8% | P=.000* |
|  | **75-84** | 76.0% | 24.0% |  | 36.6% | 63.4% |  | 58.5% | 41.5% |  |
|  | **85+** | 76.6% | 23.4% |  | 38.0% | 62.0% |  | 61.8% | 38.2% |  |
| **Country of birth** | **ISRAEL** | 76.4% | 23.6% | P<.001* | 41.4% | 58.6% | P<.001* | 60.7% | 39.3% | P=.008* |
|  | **OTHER** | 77.2% | 22.8% |  | 40.4% | 59.6% |  | 60.4% | 39.6% |  |
| **SES** | **LOW** | 80.7% | 19.3% | P=.000* | 49.0% | 51.0% | P=.000* | 66.7% | 33.3% | P=.000* |
|  | **MEDIUM** | 76.0% | 24.0% |  | 39.9% | 60.1% |  | 59.8% | 40.2% |  |
|  | **HIGH** | 75.5% | 24.5% |  | 36.5% | 63.5% |  | 57.3% | 42.7% |  |
| **Demographic sector** | **GENERAL JEWISH** | 76.8% | 23.2% | P=.000* | 39.7% | 60.3% | P=.000* | 59.8% | 40.2% | P=.000* |
|  | **CHERKESS** | 76.3% | 23.7% |  | 43.4% | 56.6% |  | 58.6% | 41.4% |  |
|  | **RELIGIOUS JEWISH** | 74.3% | 25.7% |  | 36.9% | 63.1% |  | 54.3% | 45.7% |  |
|  | **ARAB** | 81.0% | 19.0% |  | 52.3% | 47.7% |  | 70.7% | 29.3% |  |
|  | **UNKNOWN** (3.6%) | 71.1% | 28.9% |  | 37.5% | 62.5% |  | 55.1% | 44.9% |  |
| **District** | **SOUTH** | 83.5% | 16.5% | P=.000* | 42.4% | 57.6% | P=.000* | 64.0% | 36.0% | P=.000* |
|  | **CENTER** | 76.4% | 23.6% |  | 38.3% | 61.7% |  | 58.4% | 41.6% |  |
|  | **NORTH** | 75.3% | 24.7% |  | 44.4% | 55.6% |  | 61.5% | 38.5% |  |
|  | **CENTER/EAST** | 76.7% | 23.3% |  | 40.9% | 59.1% |  | 62.6% | 37.4% |  |
| **Smoking status** | **NEVER SMOKED** | 76.0% | 24.0% | P=.000* | 37.6% | 62.4% | P=.000* | 58.2% | 41.8% | P=.000* |
|  | **PAST SMOKER** | 73.1% | 26.9% |  | 32.9% | 67.1% |  | 55.1% | 44.9% |  |
|  | **CURRENT SMOKER** | 76.8% | 23.2% |  | 42.8% | 57.2% |  | 62.7% | 37.3% |  |
|  | **STATUS UNKNOWN** | 98.8% | 1.2% |  | 95.4% | 4.6% |  | 97.5% | 2.5% |  |
| **Any chronic**  **Disease** | **NO** | 89.0% | 11.0% | P=.000* | 68.4% | 31.6% | P=.000* | 79.9% | 20.1% | P=.000* |
|  | **YES** | 74.6% | 25.4% |  | 35.6% | 64.4% |  | 56.8% | 43.2% |  |
