# Supplementary material for: Adoption and Use of Telemedicine and Digital Health Services Among Older Adults in Light of the COVID-19 Pandemic: Repeated Cross-Sectional Analysis
Source: JMIR Aging. 2024 Apr 24;7:e52317. doi: 10.2196/52317 (PMC11079757; doi:10.2196/52317)
Supplement: Multimedia Appendix 2 [file aging_v7i1e52317_app2.docx]

**Appendix 2: Univariate analysis- Administrative telehealth usage – 2 categories**

|  | | **Adm- Before (0/1+)** | | | **Adm - During (0/1+)** | | | **Adm - After (0/1+)** | | |
| --- | --- | --- | --- | --- | --- | --- | --- | --- | --- | --- |
|  |  | **0 Times** | **1+ Times** | **P- value** | **0 Times** | **1+ Times** | **P- value** | **0 Times** | **1+ Times** | **P- value** |
|  |  | **%** | **%** |  | **%** | **%** |  | **%** | **%** |  |
| **Sex (male)** | **Total** | 31.0% | 69.0% | P=.000* | 25.7% | 74.3% | P<.001* | 32.1% | 67.9% | P<.001* |
|  | **FEMALE** | 30.5% | 69.5% |  | 25.5% | 74.5% |  | 31.9% | 68.1% |  |
|  | **MALE** | 31.6% | 68.4% |  | 26.0% | 74.0% |  | 32.3% | 67.7% |  |
| **Age group in 2019** | **65-74** | 31.6% | 68.4% | P=.000* | 25.7% | 74.3% | P=.000* | 32.4% | 67.6% | P=.000* |
|  | **75-84** | 27.8% | 72.2% |  | 22.9% | 77.1% |  | 29.1% | 70.9% |  |
|  | **85+** | 35.4% | 64.6% |  | 32.6% | 67.4% |  | 37.8% | 62.2% |  |
| **Country of birth** | **ISRAEL** | 34.6% | 65.4% | P=.000* | 27.6% | 72.4% | P=.000* | 35.0% | 65.0% | P=.000* |
|  | **OTHER** | 28.8% | 71.2% |  | 24.6% | 75.4% |  | 30.4% | 69.6% |  |
| **SES** | **LOW** | 39.9% | 60.1% | P=.000* | 34.3% | 65.7% | P=.000* | 41.7% | 58.3% | P=.000* |
|  | **MEDIUM** | 28.6% | 71.4% |  | 23.9% | 76.1% |  | 30.4% | 69.6% |  |
|  | **HIGH** | 27.7% | 72.3% |  | 22.2% | 77.8% |  | 27.9% | 72.1% |  |
| **Demographic sector** | **GENERAL JEWISH** | 28.6% | 71.4% | P=.000* | 24.1% | 75.9% | P=.000* | 29.8% | 70.2% | P=.000* |
|  | **CHERKESS** | 23.4% | 76.6% |  | 19.7% | 80.3% |  | 27.0% | 73.0% |  |
|  | **RELIGIOUS JEWISH** | 25.4% | 74.6% |  | 20.2% | 79.8% |  | 27.2% | 72.8% |  |
|  | **ARAB** | 55.4% | 44.6% |  | 44.2% | 55.8% |  | 55.9% | 44.1% |  |
|  | **UNKNOWN** | 27.4% | 72.6% |  | 21.5% | 78.5% |  | 29.5% | 70.5% |  |
| **District** | **SOUTH** | 26.1% | 73.9% | P=.000* | 22.6% | 77.4% | P=.000* | 27.9% | 72.1% | P=.000* |
|  | **CENTER** | 27.5% | 72.5% |  | 22.7% | 77.3% |  | 28.8% | 71.2% |  |
|  | **NORTH** | 37.2% | 62.8% |  | 31.3% | 68.7% |  | 38.7% | 61.3% |  |
|  | **CENTER/EAST** | 33.6% | 66.4% |  | 27.2% | 72.8% |  | 33.3% | 66.7% |  |
| **Smoking status** | **NEVER SMOKED** | 27.5% | 72.5% | P=.000* | 22.0% | 78.0% | P=.000* | 28.9% | 71.1% | P=.000* |
|  | **PAST SMOKER** | 21.7% | 78.3% |  | 16.4% | 83.6% |  | 22.7% | 77.3% |  |
|  | **CURRENT SMOKER** | 30.8% | 69.2% |  | 25.2% | 74.8% |  | 31.9% | 68.1% |  |
|  | **STATUS UNKNOWN** | 96.0% | 4.0% |  | 95.0% | 5.0% |  | 95.6% | 4.4% |  |
| **Any**  **Chronic**  **Disease** | **NO** | 62.8% | 37.2% | P=.000* | 57.7% | 42.3% | P=.000* | 64.0% | 36.0% | P=.000* |
|  | **YES** | 25.0% | 75.0% |  | 19.7% | 80.3% |  | 26.1% | 73.9% |  |
