# Supplementary material for: Adoption and Use of Telemedicine and Digital Health Services Among Older Adults in Light of the COVID-19 Pandemic: Repeated Cross-Sectional Analysis
Source: JMIR Aging. 2024 Apr 24;7:e52317. doi: 10.2196/52317 (PMC11079757; doi:10.2196/52317)
Supplement: Multimedia Appendix 3 [file aging_v7i1e52317_app3.docx]

**Appendix 3: Univariate analysis- Afterhours visits – 2 categories**

|  | | **Out of Hours Online Visits Count - Before (0/1+)** | | | **Out of Hours Online Visits Count - During (0/1+)** | | | **Out of Hours Online Visits Count - After (0/1+)** | | |
| --- | --- | --- | --- | --- | --- | --- | --- | --- | --- | --- |
|  |  | **0 Visits** | **1+ Visits** | **P- value** | **0 Visits** | **1+ Visits** | **P- value** | **0 Visits** | **1+ Visits** | **P- value** |
|  |  | **%** | **%** |  | **%** | **%** |  | **%** | **%** |  |
| **Sex (male)** | **Total** | 97.8% | 2.2% | P=.000* | 95.0% | 5.0% | p<.001* | 97.6% | 2.4% | p<.001* |
|  | **FEMALE** | 97.6% | 2.4% |  | 94.9% | 5.1% |  | 97.5% | 2.5% |  |
|  | **MALE** | 98.0% | 2.0% |  | 95.3% | 4.7% |  | 97.8% | 2.2% |  |
| **Age group in 2019** | **65-74** | 97.8% | 2.2% | P=0.841 | 95.0% | 5.0% | P<.001* | 97.7% | 2.3% | P=0.282 |
|  | **75-84** | 97.8% | 2.2% |  | 95.0% | 5.0% |  | 97.6% | 2.4% |  |
|  | **85+** | 97.7% | 2.3% |  | 95.5% | 4.5% |  | 97.6% | 2.4% |  |
| **Country of birth** | **ISRAEL** | 97.8% | 2.2% | P=.009* | 95.2% | 4.8% | P<.001* | 97.8% | 2.2% | P<.001* |
|  | **OTHER** | 97.7% | 2.3% |  | 94.9% | 5.1% |  | 97.6% | 2.4% |  |
| **SES** | **LOW** | 98.5% | 1.5% | P=.000* | 96.5% | 3.5% | P=.000* | 98.4% | 1.6% | P=.000* |
|  | **MEDIUM** | 97.7% | 2.3% |  | 94.9% | 5.1% |  | 97.6% | 2.4% |  |
|  | **HIGH** | 97.4% | 2.6% |  | 94.2% | 5.8% |  | 97.2% | 2.8% |  |
| **Demographic sector** | **GENERAL JEWISH** | 97.6% | 2.4% | P=.000* | 94.7% | 5.3% | P=.000* | 97.5% | 2.5% | P=.000* |
|  | **CHERKESS** | 99.2% | 0.8% |  | 98.6% | 1.4% |  | 98.6% | 1.4% |  |
|  | **RELIGIOUS JEWISH** | 97.6% | 2.4% |  | 94.5% | 5.5% |  | 97.7% | 2.3% |  |
|  | **ARAB** | 99.6% | 0.4% |  | 98.9% | 1.1% |  | 99.5% | 0.5% |  |
|  | **UNKNOWN** | 96.9% | 3.1% |  | 92.5% | 7.5% |  | 96.9% | 3.1% |  |
| **District** | **SOUTH** | 98.1% | 1.9% | P=.000* | 95.7% | 4.3% | P=.000* | 97.9% | 2.1% | P=.000* |
|  | **CENTER** | 97.4% | 2.6% |  | 94.1% | 5.9% |  | 97.3% | 2.7% |  |
|  | **NORTH** | 98.2% | 1.8% |  | 96.4% | 3.6% |  | 98.2% | 1.8% |  |
|  | **CENTER/EAST** | 97.8% | 2.2% |  | 95.0% | 5.0% |  | 97.5% | 2.5% |  |
| **Smoking status** | **NEVER SMOKED** | 97.7% | 2.3% | P=.000* | 94.8% | 5.2% | P=.000* | 97.5% | 2.5% | P=.000* |
|  | **PAST SMOKER** | 97.3% | 2.7% |  | 94.1% | 5.9% |  | 97.3% | 2.7% |  |
|  | **CURRENT SMOKER** | 98.0% | 2.0% |  | 95.6% | 4.4% |  | 97.8% | 2.2% |  |
|  | **STATUS UNKNOWN** | 99.9% | 0.1% |  | 99.7% | 0.3% |  | 99.8% | 0.2% |  |
| **Any chronic**  **disease** | **NO** | 99.0% | 1.0% | P=.000* | 97.4% | 2.6% | P=.000* | 98.8% | 1.2% | P=.000* |
|  | **YES** | 97.5% | 2.5% |  | 94.6% | 5.4% |  | 97.4% | 2.6% |  |
